# Breeding Buckwheat for Increased Levels and Improved Quality of Protein

**DOI:** 10.3390/plants10010014

**Published:** 2020-12-24

**Authors:** Zlata Luthar, Meiliang Zhou, Aleksandra Golob, Mateja Germ

**Affiliations:** 1 Biotechnical Faculty, University of Ljubljana, Jamnikarjeva 101, SI-1000 Ljubljana, Slovenia; zlata.luthar@bf.uni-lj.si (Z.L.); aleksandra.golob@bf.uni-lj.si (A.G.); 2 Institute of Crop Sciences, Chinese Academy of Agricultural Sciences, Beijing 100081, China; zhoumeiliang@caas.cn

**Keywords:** buckwheat, breeding, proteins, digestibility, flavonoids, nutritional value

## Abstract

Tartary buckwheat (*Fagopyrum tataricum* (L.) Gaertn.) and common buckwheat (*Fagopyrum esculentum* Moench) are important sources of proteins with balanced amino-acid compositions, and thus of high nutritional value. The polyphenols naturally present in Tartary buckwheat and common buckwheat lower the true digestibility of the proteins. Digestion-resistant peptides are a vehicle for fecal excretion of steroids, and in this way, for bile acid elimination and reduction of cholesterol concentrations in serum. Buckwheat proteins are more effective compared to soy proteins for the prevention of gallstone formation. Tartary and common buckwheat grain that contains appropriate amounts of selenium-containing amino acids can be produced as functional food products. The protein-rich by-products of buckwheat are a good source of bioactive substances that can suppress colon carcinogenesis by reducing cell proliferation. The grain embryo is a rich source of proteins, so breeding buckwheat with larger embryos is a possible strategy to increase protein levels in Tartary and common buckwheat grain. However, chemical analysis of the grain is the most relevant criterion for assessing grain protein levels and quality.

## 1. Introduction

Tartary buckwheat (*Fagopyrum tataricum* (L.) Gaertn.) and common buckwheat (*Fagopyrum esculentum* Moench) are traditionally grown in the Himalayas, south-east Asia, and central and eastern Europe. Buckwheat is a low input plant and is suitable for cultivation in mountainous regions. In some parts of the world, buckwheat is an important component of the diet, and thus an important source of carbohydrate, protein, and secondary metabolites in the human diet [1,2,3,4,5]. Buckwheat noodles are a popular dish in Japan, and traditionally in some rural areas of China, Korea, and Bhutan, and in regions on the southern slopes of the European Alps, including Slovenia, Italy, Switzerland, and France.

In Slovenia, bread made with Tartary and common buckwheat is used traditionally, and its consumption is again becoming popular [5]. Buckwheat groats (i.e., husked buckwheat grain) are traditionally produced and consumed in central and eastern Europe (i.e., Slovenia, Croatia, Poland, Ukraine, Russia), and can be used in a similar way to rice [5,6].

While breeding of common buckwheat has been widespread in all buckwheat growing areas, Tartary buckwheat is a specialty crop that is appreciated in less favorable environmental conditions. Until recently, Tartary and common buckwheat breeding were designed to achieve high yields and resistance to less favorable environmental conditions. More recently, special attention has been paid towards the optimization of the nutritional quality parameters of Tartary buckwheat and common buckwheat, which has included high protein content and optimal amino-acid composition of the proteins. Here, we review the possibilities to develop Tartary and common buckwheat for grain with higher protein levels with improved amino-acid composition.

## 2. Protein Levels in Buckwheat Grain and Milling Fractions

Protein levels in buckwheat grain are relatively low (~12%) compared to leguminous plants (soybean meal, ~51%), although they are higher compared to most cereals [7]. It is possible to concentrate proteins by milling and separation of the fractions of common buckwheat. The milling fractions with the highest protein levels is the bran, with ~30% protein in terms of dry matter [8]. The highest levels of the flavonoid rutin were also detected in the same milling fraction. The lowest protein levels are seen for the flour fraction, with only 4.4% [8].

By milling Tartary buckwheat, bran with 25% protein can be obtained, with the flour with 10% protein [9,10]. Thus, by adapting the milling methods, it is possible to obtain products for diverse uses and nutritional values.

## 3. Quality of Buckwheat Protein Compared to Soybean and Cereals

According to Eggum [7], common buckwheat contains 5.1% lysine in its protein fraction (i.e., 5.1 g/16 g N). For comparison, lysine levels in the protein fractions measured by the same method were 2.6% for wheat, 2.8% for maize, and 3.7% for barley (Table 1). Lysine levels for the protein in soybean meal are 6.0% and the same in fava bean. Due to the high levels of lysine, and the well-balanced levels of the other amino acids, the biological value of buckwheat proteins is 93%. From the same type of experiments with rats, soybean meal proteins have a biological value of 68%, compared to that of pork protein, at ~84%. Despite the higher lysine levels in soybean and fava bean, the buckwheat protein is superior, with the lower biological value of the soybean and fava bean proteins due to their lower levels of sulfur-containing amino acids (Table 1) [7].

These low levels of sulfur-containing amino acids in beans means that quality is compensated for by quantity. However, according to Eggum [7], high quantities of protein are not an advisable solution to the problem of this less balanced amino acid composition in beans. These bean proteins that contain amino acids with lower nutritional quality is the superficial amount of nonessential amino acids. This superficial amount is used by an organism only as a source of energy. However, this represents a relative waste of energy, as proteins are not a good energy source [7].

The distribution of sulfur in grain can be used as a marker of protein distribution among different grains and within the different parts of the grain [11,12]. Recently, the use of microparticle-induced X-ray emission (micro-PIXE) has improved the detection of sulfur content and its allocation to the different structures of buckwheat grain, which might serve for estimation of the relative levels of proteins containing sulfur amino acids in the cotyledons and the aleurone [13,14,15].

Seed storage proteins in buckwheat are mainly water and saline soluble, in contrast to gluten-containing cereals [16,17,18]. Buckwheat grain proteins consist of up to 50 to 60% albumins and glutelins [19]. Buckwheat samples also have high concentrations of lysine, although threonine and sulfur-containing amino acids are the first limiting amino acids in regard to the need for amino acids in human nutrition [20,21,22,23,24].

The amino acids serine, proline, glycine, histidine, and arginine, obtained after in-vitro digestion of buckwheat bran appears to have high cytotoxic effects on cultured colon cancer cells. The final cytotoxic effects arise from not only the levels of bioactive substances, but also as a function of the synergistic actions among these. Thus, buckwheat protein-rich by-products are a good source of bioactive substances [25,26,27,28,29]. However, in-vitro antiproliferative effects on cancer cells do not always predict health-promoting effects, as the impacts must also be studied and confirmed in vivo. Some literature data [26] have stressed the need for a complex evaluation of the biological activities of buckwheat metabolites on intestinal health. Consumption of buckwheat protein extracts has been shown to slow mammary carcinogenesis in rats, which was connected with muscle hypertrophy and reduced hepatic triglyceride concentrations [30,31,32,33].

In selenium-enriched buckwheat plants, the resulting proteins are interesting in terms of their selenium content [34]. It is feasible to increase the selenium concentration in Tartary and common buckwheat grain by fertilizing them with selenium compounds so that they become a good source of seleno amino acids [35,36]. In selenium-enriched buckwheat grain, 39% of the selenium is present as water-soluble selenium, such as in peptides, proteins, and other selenium compounds; also, 93% of this water-soluble selenium is selenomethionine [37], which is one of the most bioavailable forms of selenium for animals and humans.

The selenium content in Tartary buckwheat under ambient UV-B radiation is higher compared to common buckwheat. Tartary buckwheat can grow at higher altitudes than common buckwheat. In mountain areas, the UV-B radiation is more intense, and selenium might protect the plants from strong UV-B radiation. Enhanced UV-B radiation increases the selenium content in the vegetative parts of common buckwheat without selenium treatment [37]. In addition, higher selenium levels have been shown for selenium-treated Tartary buckwheat compared to selenium-treated common buckwheat [38]. The grain of selenium-treated hybrid buckwheat also shows increased accumulation of selenium when exposed to reduced UV radiation, compared to plants exposed to ambient UV radiation levels [39].

With regards to selenium and flavonoids, there are similar effects of UV-B radiation on flavonoid content in buckwheat. Tartary buckwheat contains more flavonoids than common buckwheat [40]. Kreft et al. [41] studied the impact of UV-B radiation on rutin content and reported that this was higher in plants exposed to ambient levels of UV-B radiation, compared to plants grown under reduced UV-B radiation.

## 4. Buckwheat Protein Digestibility

Proteins from Tartary buckwheat and common buckwheat have low digestibility [42,43]. The polyphenols that are naturally present in Tartary buckwheat and common buckwheat, including rutin and quercetin, lower the true digestibility of the proteins [43,44,45,46]. Ikeda et al. [47] reported that phenolic substances have significant inhibitory effects on in-vitro peptic and pancreatic digestion of globulin, and thus Tartary buckwheat and common buckwheat secondary metabolites might have an impact on protein digestibility [48]. Considerable interactions between polyphenols and proteins are observed after hydrothermal treatment [45]. In buckwheat, the situation is similar to millet, where the protein digestibility is slower compared to other cereals, potentially because of the binding of polyphenolics to proteins [49].

The interactions between proteins and phenolic substances slow down the digestion of proteins in the small and large intestine. However, microbial fermentation in the colon can enhance the digestibility of proteins blocked by polyphenols in hydrothermally processed buckwheat [44,45]. Digestion-resistant peptides are important for the fecal excretion of steroids. Buckwheat proteins prevent gallstone formation more strongly in comparison to soy proteins. They might also slow mammary carcinogenesis by lowering serum estradiol and suppress colon carcinogenesis by reducing cell proliferation [50,51,52,53,54,55,56].

It would be expected that breeding Tartary buckwheat and common buckwheat for lower grain polyphenols content will enhance the nutritional value of the proteins; however, this might instead be unfavorable for the beneficial impact of polyphenol-protein complexes for preventing diseases [45,51].

## 5. Bioactivity of Buckwheat Peptides

Buckwheat peptides can be obtained by the fermentation of buckwheat grain or sprouts.

The buckwheat grain is the source of biofunctional peptides [28,57,58,59,60,61], which are fermented in the process of malting [62] and during the preparation of buckwheat beer or distillation products [63,64].

Sprouts from Tartary and common buckwheat grain are prepared to produce buckwheat vegetable, which is rich in flavonoids and mineral elements [65,66,67]. Sprouts can be further processed by fermentation, and valuable products with reported blood-pressure-lowering effects can be obtained [68,69,70]. Purified buckwheat proteins and peptides can be used due to their beneficial effect in atherosclerosis [71,72] and hypercholesterolemia [73,74,75,76].

There are possibilities to develop novel buckwheat products based on the functional and technological values of buckwheat proteins and peptides [74,75,77,78,79,80]. The properties of buckwheat proteins and isolates can be altered by thermal and ultrasound treatment. Maillard reaction can change the proteins to modify their allergenicity [81,82,83]. Another method to obtain novel buckwheat protein-rich food products is to fractionate buckwheat grain by milling [84]. However, none of these methods appears to be satisfactory for sufficient removal or inactivation of allergenic buckwheat proteins in the products. This is a pity because some buckwheat products are used for gluten-free products, and some customers might have multiple food intolerance syndromes [85,86,87]. Tartary and common buckwheat, alone or in combination with rice or corn, are used in the development of gluten-free food products [88].

Buckwheat leaf flour and grain can be used to produce protein isolates [89,90,91,92,93]. However, the process should include the removal of polyphenolic substances [94]. On the other hand, tannin-protein complexes can be used in buckwheat products due to their radical scavenging effects; these complexes can provide effective radical sinks and modify protein digestibility [17,45,95,96].

## 6. Buckwheat Breeding for High Protein Levels: Prospects for the Future

**Figure 1:**
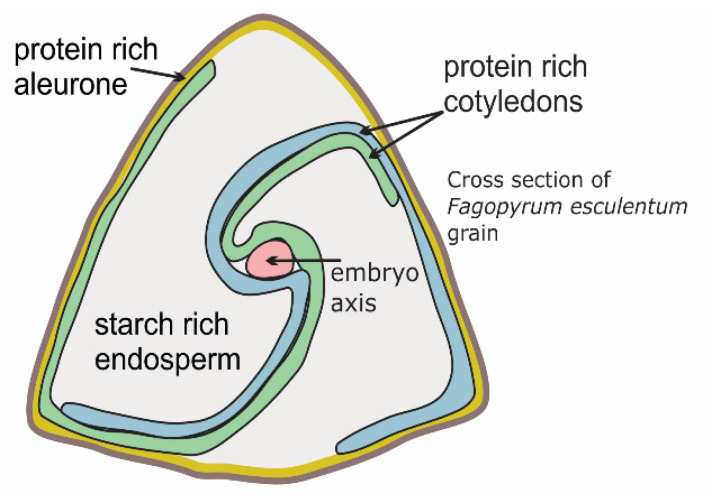
Cross-section of a common buckwheat grain.

Buckwheat grain is rich in high-quality proteins and contains well-balanced amounts of essential amino acids (i.e., lysine, methionine, cysteine, tryptophan). In addition, common buckwheat is important as a nectariferous plant, and both Tartary and common buckwheat are pharmaceutical plants [97,98,99,100]. In recent buckwheat breeding programs, particular properties have been investigated, such as high grain yield, frost resistance, increased protein content, reduced allergenic protein content [97]. People with multiple allergies can also develop an allergy when eating buckwheat [85], which can be caused by low-molecular-weight proteins in the grain embryo (i.e., 18–29 kD) [84,100,101,102,103,104,105]. However, there have not been any reports on the allergenicity of endosperm proteins and of proteins with diverse molecular weights in the aleurone. Besides the embryo, the aleurone is the main grain tissue with a high protein content (Figure 1). In the preparation of buckwheat food products, it is important to understand how endosperm and embryo cells are damaged under the technological processes to make the content of the cells available for intestinal digestion [106,107]. Temperature treatments and intestinal microbial activity are other factors of importance in the production of buckwheat foods with health promoting effects [68,108,109,110,111]. It would be expected that breeding Tartary buckwheat and common buckwheat for larger embryo and thicker aleurone will enhance the amount of the grain proteins. Genetic variability of embryo size has been reported to have an impact on the chemical composition of grains, as reported for barley and genus *Avena* [112,113]. Microscopic analyses of buckwheat grain might represent a method for screening for buckwheat breeding, in terms of a larger embryo and aleurone, and thus also for higher protein levels in the grain [15] (Figure 1).

**Figure 2:**
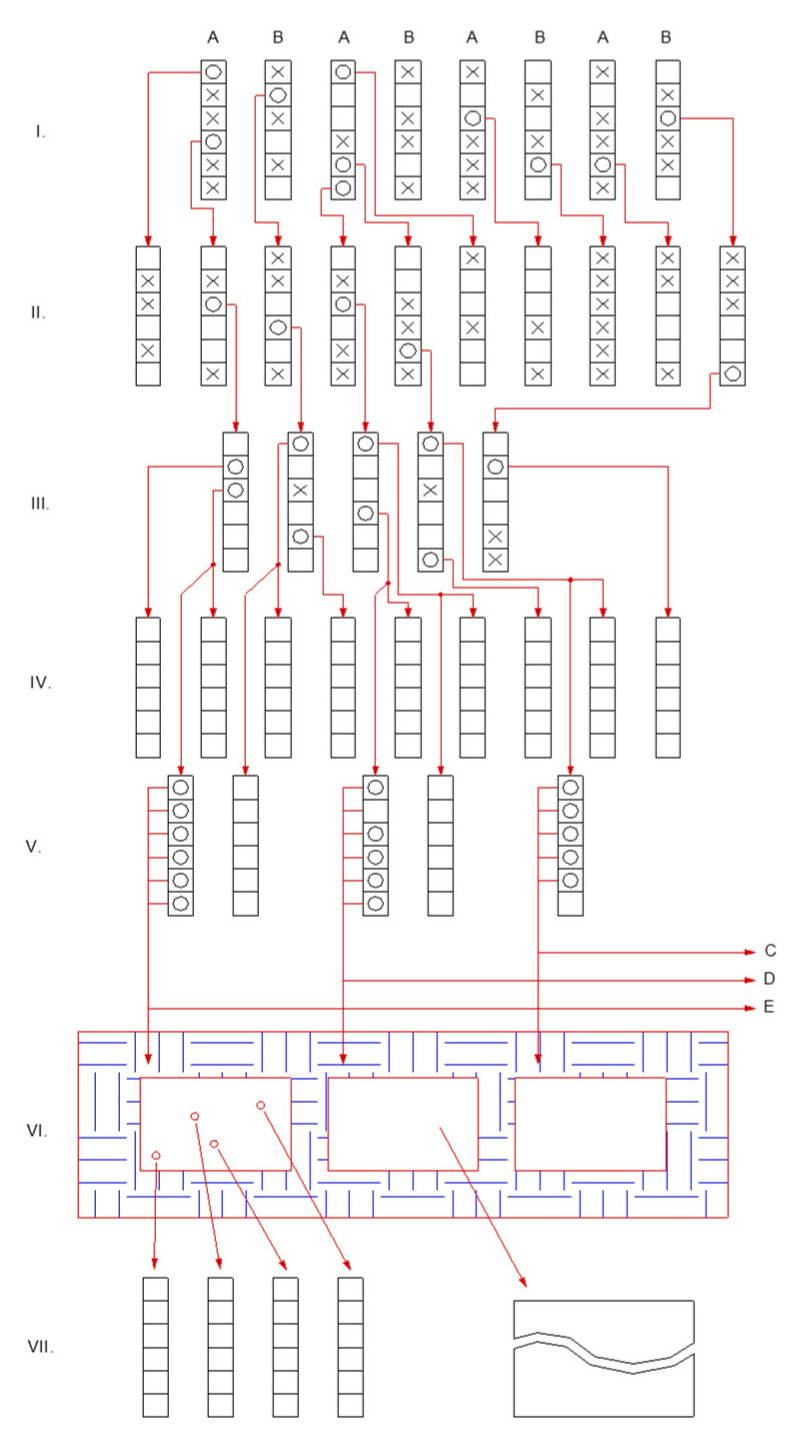
Stages of buckwheat breeding over time. The Roman numerals (**I**–**VII**) indicate the years (generations) of the breeding. The starting material in the first year is two-parent varieties or populations, indicated as A and B. Both parents are sown in parallel rows. For one parent, after opening the first flower on each plant, the plants with thrum flowers are removed. In the other parent, the plants with pin flowers are removed. So in generation (**I**), only fertilization between A and B flowers is possible, and not fertilization among the plants within each parent. Each square symbolizes a plant, and the plants marked with “x” are removed from breeding, while the plants marked with “o” are selected for further propagation and breeding. Groups of plants with empty squares are those that are used for testing according to the phenotype in the field trial, but not for further propagation or breeding. The best-selected plant material is isolated and taken forward for further propagation or breeding or for submission to official variety testing. In generation (**VI**), the plants are sown in isolation, for evaluation of yield and for propagation. The plant groups C, D, and E are taken on to the first field tests on larger isolated plots.

At the same time, there is greater protein polymorphism within varieties of common buckwheat than between varieties [114]. This high polymorphism can be exploited in breeding, although like globulin, most of the proteins show single Mendelian gene inheritance, with a codominant expression of multiple alleles, which complicates plant breeding [115]. Cross-fertilization of common buckwheat has an impact on the breeding approach. The suggested procedure for common buckwheat breeding, according to Kreft [116], is presented in Figure 2. Common buckwheat is a cross-pollinating plant. In the field breeding experiments, diploid common buckwheat must be in physical isolation (in tents or glasshouses), so uncontrolled pollination is prevented in common buckwheat breeding (Figure 2). Such isolation is not needed in the breeding of self-compatible Tartary buckwheat varieties.

Under Slovenian and similar climatic conditions, it is possible to grow two generations of buckwheat in 1 year. Under these Slovenian conditions, the first generation of the year is sown at the beginning of May and harvested in mid-July. The second generation is thus sown about a week after the harvest of the first generation and is harvested in early October. The second-generation (Figure 2, II) can theoretically be uniform if the parent plants are homozygous. However, in common buckwheat populations or varieties the plants are never homozygous, because the populations or varieties are obligatory cross-pollinated. Therefore a certain selection is already feasible and reasonable in the second generation (Figure 2, II). Isolation is possible by means of greenhouse compartments or tents, which prevents insects from visiting from outside, while bumblebees in each compartment can pollinate the plants. Another method of isolation is to use a tetraploid buckwheat area between the diploid breeding material lots. It has been shown [116] that 12 m to 20 m of such isolation is sufficient to prevent undesirable plant crossings [116,117,118]. The pollination of diploid plants with pollen grains from tetraploid plants or vice versa, results in aborted or sterile seeds.

The number of proteins per seed can be estimated by screening the embryo size in cross-sections of grain [112,113]. However, the more accurate methods are by chemical analyses. The problem is that for chemical analysis, some of the seeds are milled, and hence, they are lost from further seed propagation of the respective sample. It is thus necessary to use as small a sample for protein analyses as possible or to cut off the upper third of the seed only, which covers part of the cotyledons and aleurone. The remaining part of the seed can then be sown for the next generation.

Breeding programs include the development of adapted high-yield varieties that have other good agronomic characteristics, such as high levels of rutin, the very high biological value of the proteins, or reduced levels of antinutritional factors and zero allergenic protein [19,119,120]. In the future, it would make sense for buckwheat breeding programs to include genotypes with larger endosperm and smaller embryo as an option to reduce allergenic proteins to produce specific low allergenicity buckwheat varieties.

A recently published draft sequence of genes of the common buckwheat [121,122] has allowed the use of genomic selection for quantitative traits based on DNA markers, as well as other methods related to the quality of proteins [123]. Furthermore, the availability of such information facilitates the identification of markers for the identification of qualitatively useful genes [121,124]. A major advantage of genomic selection is that, in contrast to the conventional marker-assisted selection, it is suitable for improving quantitative properties managed by multiple quantitative trait loci. Genomic selection and use of DNA markers in breeding buckwheat protein quality are feasible only at the initial stages [125,126,127]. Rapid advances in genomic technology will certainly improve genomics-based breeding for buckwheat quality in the near future [128].

## 7. Conclusions

Buckwheat is a low-input plant that is suitable for cultivation in mountainous regions. Recently, special attention has been paid to optimizing the nutritional quality parameters of Tartary buckwheat and common buckwheat, including its high protein content and optimal amino acid composition. The distribution of sulfur in the grain can be used as a marker of protein distribution among the different grains and within the different parts of the grain. The use of micro-PIXE has improved the detection of sulfur and its distribution in the grain, which can be used to estimate the relative content of proteins with sulfur amino acids in the cotyledons and aleurone.

It is possible to increase the selenium concentration in buckwheat grain by fertilizing plants with selenium compounds, making them a good source of selenoamino acids. In selenium-enriched buckwheat grain, 39% of selenium is present as water-soluble selenium—e.g., in peptides, proteins, and other selenium compounds. In addition, 93% of this water-soluble selenium is selenomethionine, which is one of the most bioavailable forms of selenium for human nutrition.

Rutin, quercetin, and other polyphenols, that are naturally present in buckwheat, reduce the digestibility of the proteins. Phenolic substances have significant inhibitory effects on the in vitro digestion of globulin in the intestine, so secondary metabolites of buckwheat can have an influence on the digestibility of proteins. Significant interactions between polyphenols and proteins are observed after hydrothermal treatment. In buckwheat, protein digestibility is slower compared to other cereals, possibly due to the binding of the polyphenols to the proteins. The interactions between proteins and phenolic substances slow down proteins’ digestion in the small and large intestine. However, microbial fermentation in the large intestine can improve the digestibility of proteins blocked by polyphenols in hydrothermally treated buckwheat. Digestion-resistant peptides are important for the fecal excretion of steroids. The excretion of bile acids reduces the cholesterol concentration in serum. Buckwheat proteins are more effective at preventing the formation of gallstones than soya proteins. There are opportunities to improve protein content and quality through buckwheat breeding and to develop novel buckwheat products based on buckwheat proteins and peptides’ functional and technological value.

## Figures and Tables

**Table 1:** Contents of essential amino acids and biological value of various protein sources (adapted from Eggum [7]).

Protein Source	Amino Acid (g/16 g N)			Biological
	Lysine	Methionine	Cysteine	Value (%)
Buckwheat	5.09	1.89	2.02	93.1
Wheat	2.55	1.81	1.79	62.5
Soybean	5.99	1.61	1.59	68.4
Fava bean	6.04	0.94	1.44	51.1
Egg	6.79	3.09	2.38	99.3
Pork	7.99	2.85	1.14	84.3
